# Pyrethroid insecticides susceptibility profiles and evaluation of L1014F kdr mutant alleles in *Culex quinquefasciatus* from lymphatic filariasis endemic communities

**DOI:** 10.1038/s41598-023-44962-2

**Published:** 2023-10-31

**Authors:** Martina Anurika Okafor, Ndifreke Daniel Ekpo, Kenneth Nnamdi Opara, Nsima Ibanga Udoidung, Farid S. Ataya, Clement Ameh Yaro, Gaber El-Saber Batiha, Athanasios Alexiou, Marios Papadakis

**Affiliations:** 1 Department of Animal and Environmental Biology, University of Uyo, Uyo, Akwa Ibom State Nigeria; 2 Department of Biochemistry, College of Science, King Saud University, PO Box 2455, 11451 Riyadh, Saudi Arabia; 3 Department of Pharmacology and Therapeutics, Faculty of Veterinary Medicine, Damanhour University, Damanhour, AlBeheira 22511 Egypt; 4 Department of Science and Engineering, Novel Global Community Educational Foundation, Hebersham, NSW 2770 Australia; 5 AFNP Med, 1030 Wien, Austria; 6 Department of Surgery II, University Hospital Witten-Herdecke, University of Witten-Herdecke, Heusnerstrasse 40, 42283 Wuppertal, Germany

**Keywords:** Ecology, Molecular biology, Diseases

## Abstract

This study investigated the dynamics in pyrethriod resistance and the presence/frequencies of L1014F knockdown resistance mutant allelles in *Culex quinquefasciatus* vector populations from Uruan Local Government Area of AkwaIbom State, Southern Nigeria between the months of March and November, 2021. Uruan LGA is among the endemic LGAs for lymphatic filariasis in AkwaIbomState. Female *Anopheles* mosquitoes from Eman Uruan, Ituk Mbang and Idu Uruan were exposed to permethrin, deltamethrin and alphacypermethrin in CDC insecticide coated bottles for susceptibility bioassay following standard protocols. The mosquitoes were obtained as aquatic forms from the study sites and reared under laboratory conditions to adults. The adult mosquitoes were used for this study. All the mosquitoes used for the insecticide susceptibility bioassay were morphologically identified. Standard Polymerase chain reaction (PCR) was used for authenticating the *Culex quinquefasciatus* species. A portion of the vgsc (917 bp) gene spanning the entire intron and the exon containing the L1014F mutation associated with knockdown resistance (*kdr*) in the vectorswere amplified using Allele-SPECIFIC POLYMERASE CHAIN REACTION (AS-PCR) in order to detect target site insensitivity in the vectors from the study sites. Results obtained revealed that vectors from all the study sites were resistant to permethrin insecticide (mortality rate: 18–23%). Suspected resistance (mortality rate: 90–93%) to deltamethrin and low resistance (mortality rate: 82–85%) to alphacypermethrin insecticides were detected. knockdown was more rapid with deltamethrin and alphacypermethrin than with permethrin across the study sites considering their KDT_50_ and KDT_95_. The frequency of the resistant phenotypes ranged from 35.14 to 55.3% across the study sites with a net of 45.1% resistant phenotype recorded in this study. The 1014F allelic frequency calculated from Hardy–Weinberg principle for vector populations across the study sites ranged from 0.500 (50.00%) to 0.7763 (77.63%). All populations witnessed significant (*p* < 0.05) deviations from Hardy–Weinberg equilibrium in the distribution of these alleles. The findings of this study show that there is a tendency to record an entire population of resistant vectors in this study area over time due to natural selection. The public health implication of these findings is that the use of pyrethroid based aerosols, coils, sprays, LLITNs and others for the purpose of controlling vectors of lymphatic filariasis and other diseases may be effort in futility.

## Background of study

One of the most important evolutionary phenomenon for researchers in recent times is insecticide resistance. A large number of infectious and parasitic diseases that threaten public health are transmitted by pathogens that are vectored by a diverse groups of insects^1,2^. Among different insect vectors, mosquitoes are responsible for many dreaded tropical diseases including lymphatic filariasis^3^. Lymphatic filariasis is endemic in tropical and sub-tropical areas of the world. I|n Africa, 34 countries are endemic; and Nigeria is believed to bear the highest burden of the disease, with an estimated 80–120 million people at risk^4^. Disease control programmes in many countries of the world today have experienced collapse due to chemical over-reliance or overuse which induces resistance in insect vectors^5^. Since the resistance of insects to insecticides was first described^6^, it has emerged as a major topic for research and discussion in public health, because its presence in disease vectors is one of the major obstacles to the reduction of the burden ofvector-borne diseases in endemic countries^7^.

The mosquitoes, *Culex pipiens*and *Culex quinquefasciatus* acts as vectors of several pathogens in both tropical and temperate environments, and in many tropical/ sub-tropical regions, *C. quinquefasciatus* is the primary vector of lymphatic filariasis^8–11^. Culex species are also responsible for a high nuisance problem^12,13^. The vector displays a variety of breeding habitats, including swamps, drains, pit latrines, and permanent or semi-permanent stagnant water bodies, full of organic matter^14,15^, commonly found within and around African sites. The nature of the Akwa Ibom environment promotes the spread of the disease. Contaminated water in pit latrines, septic tanks and drains equally constitute good breeding habitats for *Culex quinquefasciatus*^16–18^.

In the absence of effective vaccines against most of the *Culex* transmitted pathogens, the best strategy to avoid transmission relies on chemical control of the mosquitoes^19^. As observed^19,20^, vector control is a key component in disease management. Integrated Vector Management (IVM) is a supplemental strategy recommended to stop the spread of lymphatic filariasis and other co-endemic mosquito-borne diseases like malaria^19^. Six classes of insecticides: Organochlorides, Organophosphates, Carbamates, Pyrethroids, Pyrroles, and Phenyl pyrazoles are recommended for use against adult mosquitoes^21^. Pyrethroids are still one of the most important classes of insecticides used against mosquitoes—especially for treated bed nets and as aerosolised sprays^22^. The effectiveness of insecticide-based vector control is threatened by the increased insecticide resistance which has been detected across the globe^23,24^. The increased resistance to pyrethroids is a major bane since this insecticide is the most common active ingredient used to control adult mosquitoes through indoor sprays, outdoor fogs and treated bed nets^25^.

The voltage-gated sodium channel (VGSC) is the target site for pyrethroid insecticides^26^. According to^26^, it is located in the insect’s nervous system. Mosquitoes resistant to pyrethroid insecticides exhibit some modification of their VGSC, due to a mutation of the gene encoding this protein^26^. The contributions of the L1014F allele to the insecticide resistance landscape of mosquitoes have been widely reported^27–30^. Nevertheless, it has been documented that this gene is recessive and as such, the mutation can only be phenotypically expressed at homozygous state. Insecticide resistance-related mutations in the vector populations may be driven to even higher frequencies, consequently decreasing the efficacy of treatment. Increased resistance to insecticides threatens the efficacy and sustainability of insecticide-based anti-vector interventions which mitigate the burden of mosquito transmitted diseases in endemic regions^29^. Monitoring of resistance patterns and understanding the underlying mechanisms is vital for extending the lifespan of currently available insecticides as well as for planning more effective vector control programmes^31^. This is because the vulnerability of mosquito vectors to control measures is very focal and local^32^, varying by mosquito species and being influenced by local environmental factors. It is advocated that before an evidence based policy direction is designed for the implementation of integrated vector management in Lymphatic filariasis control, insecticide resistance monitoring must be given a very wide spread within a geographical area^27,33^. Unfortunately, data on pyrethroid resistance profile are still very limited, highly insufficient and difficult to consolidate^27^ and for this reason, designing evidence based malaria vector control policies by the National Malaria Elimination Programme (NMEP) of the Federal Government of Nigeria may be a mirage^28^.This work aimed at assessing the susceptibility of *Culex quinquefasciatus* vectors from Uruan LGA in Akwa Ibom State, Nigeria to deltamethrin, alphacypermethrin and permethrin insecticides; and detection of L1014F knockdown resistance mutations in the vector populations.

## Methods

### Study area

**Figure 1:**
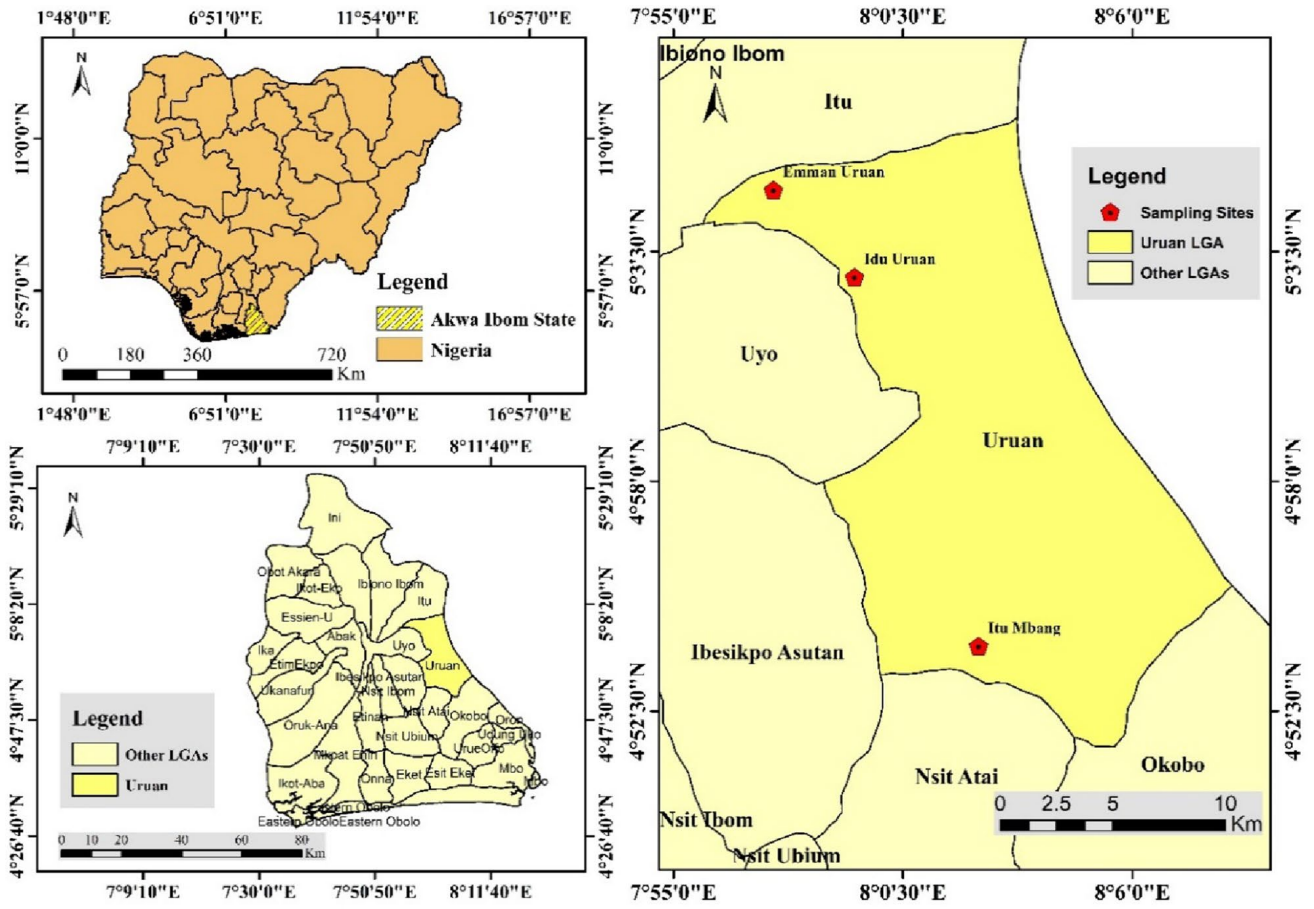
Map showing location of study sites.

The study was carried out in Uruan Local Government Area (LGA) of Akwa Ibom State, Nigeria between the months of March and November, 2021. Uruan LGA is among the endemic LGAs for lymphatic filariasis (LF) in Akwa Ibom state. The area lies within 7°96′89.41″ N (Longitude) and 5°023′4.90″ E (Latitude) (Fig. 1). The area is marked by grassy vegetation with randomly moderate tall trees; its topography is of undulating type, with stagnant water, ponds and a sluggish water body. The rich coastal plains support the cultivation of crops like Cassava, Maize and others. The study sites selected for this study were villages in Northern, Central and Southern Uruan Districts including Emman Uruan, Idu Uruan and Ituk Mbang respectively. All the study sites were characterized by thick bushes, swamps, forest, clustered residential buildings, water logs as well as shallowly dug wells are vastly distributed. All these present good breeding ground(s) for mosquitoes; and such breeding sites were identified.

### Larvae collections

Survey was done in the study area to establish presence of potential breeding sites of *Culex* mosquitoes. Various sampling sites were identified to include gutters, ponds, stagnant water, rain puddles, among others. Larval identification was based on its characteristics^34^. *Culicine* larvae were identified by their angular position. They were carefully collected with a 350 ml dipper and transferred into 10 L plastic jerrycan which were loosely capped to allow aeration. The water samples containing larvae and other aquatic stages of *Culicine* were then transported to the USAID/PMI/VECTORLINK Malaria Research Laboratory/ Insectary in the Department of Animal and Environmental Biology, Faculty of Science, University of Uyo for rearing.

### Laboratory rearing of mosquitoes

In the laboratory, the larvae were transferred into a rectangular plastic container (with a capacity of 20 L) and covered with a net of tiny meshes to allow aeration and also prevent the adult that emerged from escaping. The larvae were fed with a blend of cabin biscuit and yeast prepared by grinding two pieces of cabin biscuits and 10 tablets of yeast together. Following emergence of the adults, they were aspirated with aspirators into adult mosquito cages. The adult populations were maintained by feeding them with ten percent (10%) sugar solution.

### Isolation of female Culex quinquefasciatus mosquitoes from the pool of other Culicines

This was instrumental in determining the Discriminating Doses (DD) and the Diagnostic Time (DT) of insecticide used for the susceptibility bioassay. Adult Female *Culex quinquefasciatus* mosquitoes were successfully identified and isolated from other *Culicines* (including males) using morphological keys^34^, and a dissecting microscope (Olympus, USA). Females were identified by their non-feathery (Pilose) antennae and their slender palpi—about half the length of the proboscis^35^. A characteristic carton brown colour, the presence of thick half-moon shaped pale bands on the abdominal terga and a completely pale ventral abdominal region, were the striking morphological features used to successfully identify and isolate *Culex quinquefasciatus* mosquitoes from the pool of other *Culicines.*

### Insecticide susceptibility bioassay on Culex quinquefasciatus vector populations

Insecticide susceptibility bioassay was carried out according to standard protocol as outlined by the CONUS Manual for Evaluating Insecticide Resistance in Mosquitoes Using the CDC Bottle Bioassay Kit (https://www.cdc.gov/zika/vector/insecticide-resistance.html., 2021)^36^. It involved the use of specially designed CDC bottles. The bottles were first coated with pyrethroid insecticides including permethrin (43 µg/bottle), deltamethrin (0.75 µg/bottle) and alphacypermethrin (0.75 µg/bottle). A 2–5 days old and non-blood fed female *Culex quinquefasciatus* mosquitoes were used in the CDC bottles for this test. Twenty-five (25) mosquitoes were first aspirated from the population in the cage and gently blown into the control bottle coated with acetone only. Next, one hundred mosquitoes, from that same population, were aspirated in batches of 25 into each of the four insecticide coated bottles. The timer was started, and the number of dead and/or alive mosquitoes at time zero were counted and recorded. The number of live and or knocked down mosquitoes were recorded at 15 min, 30 min, 45 min and 60 min respectively. A mosquito was considered knocked down if it was unable to stand or fly in a coordinated way. Knock down at the diagnostic time (DT) for each insecticide (30 min for Permethrin and 60 min for Alphacypermethrin and Deltamethrin) was used as an indicator of the population susceptibility. Where knock down (KD) between 3 and 10% was observed in the controls, the percentage mortality was recalculated for the insecticide tested, using Abotts’s formula^37^. Test results were discarded where the mortality in the control bottle at the end of the test was more than ten percent (10%). Each round of the test (conducted per site) involved four replicates for each of the three different insecticides; and one control experiment. At least three hundred and seventy-five (375) 2–5 days old, non-blood-fed active adult female *Culex quinquefasciatus* mosquitoes were randomly sampled per site and subjected to this test at each round. The same procedure was repeated for all the three (3) study sites covered within the study area. The susceptibility status of mosquito populations tested with the CDC bottles was determined according to the CDC criteria^38,39^, as follows:

Mortality rates between 98 and 100% at DT, indicated full susceptibility*.* Mortality rates between 90 and 97% at DT, indicated possible resistance to be confirmed Mortality rates < 90%, indicated a population resistant to the tested insecticide.

### Species authentication/profiling

Using a random cluster sampling procedure, one hundred and thirteen (113) random samples were drawn from both susceptible population (those that didn’t survive the test) and resistant population (those that survived the test) of mosquitoes across the study sites and subjected to species authentication/ profiling which targeted the amplification of fingerprint genes for each species of *Culex* mosquito in the population. DNA were extracted from each of the mosquitoes and subjected to the PCR assay. The PCR product were electrophoresed and an established diagnostic band size was used in identifying and authenticating the species following the method of Smith and Fonseca^40^. Briefly genomic DNAs were extracted from each mosquito using the Jena Bioscience Blood-Animal-Plant DNA preparation kit following the manufacturer's instructions. One microlitre (1 μl) of the DNA extract was taken using micropipette and transferred into a separate tube containing 25µL PCR master mix 2X (Dremtaq green PCR Master Mix (THERMOSCIENTIFIC), made up optimized Dream Taq buffer, 4 mM MgCl_2_, 0.4 mM each of, dNTPs; (dATP, dCTP, dGTP, dTTP), Taq polymerase, 1.0 µM each of diagnostic primers and nuclease free water to make 50µL of PCR reaction mix. Two primers: ACEquin (5′-CCTTCTTGAAT GGCTGTGGCA-3′), and B1246s (5′-TGGAGCCTCCTC TTCACGG-3′) were used to amplify at 274 bp diagnostic fragment of the entire extracted DNA of *Cx quinquefasciatus* according to the method of^40^. A Perkin Elmer 480 Thermal Cycler (Perkin Elmer Cetus, Norwalk, Connecticut, USA) was used for the PCR amplification process. Thermocycler conditions consisted of an initial denaturation step of 95 °C for 2 min; annealing at 55 °C for 1 min, and extension at 72 °C for 45 s; followed by 33 cycles of PCR with each cycle consisting of denaturation at 94 °C for 30 s, annealing at 52 °C for 1 min and extension at 72 °C for 45 s; and a final cycle consisting of denaturation at 94 °C for 30 s, annealing at 52 °C for 1 min, and elongation at 72 °C for 5 min making a total of 35 cycles. A control, which contained no DNA template was included for each set of reaction.

### Allele-specific PCR (AS-PCR) assay to detect knock down resistance (kdr) mutation in Culex vectors from the study area

The one hundred and thirteen (113) mosquitoes randomly sampled from both susceptible population (those that didn’t survive the test) and resistant population (those that survived the test) were used for the AS-PCR Assay. The PCRs were performed to detect L1014F kdr mutant alleles in each of the mosquito following the protocol of^41^ with minor modifications as described by^42^. Briefly, four primers (Cq1, Cq2, Cq3 and Cq4) were used for this purpose. Two primers [Cq1 (Forward), 5′-GTGGAACTTCACCGACTTC-3′ and Cq2 (Reverse), 5′-GCAAGGCTAAGAAAAGGTTAAG-3′] were used to amplify the fragment of sodium channel gene containing the L1014F kdr mutant alleles. The other two primers [Cq3 (Forward), 5′-CCACCGTAGTGATAGGAAATTTA-3′ and Cq4 (Forward), 5′-CCACCGTAGTGATAGGAAATTTT-3′] were allele specific primers used in genotyping of L1014F [Cq3] and L1014L [Cq4] alleles by allele-specific PCR assay (AS-PCR). These allele specific primers (Cq3 and Cq4) differed only at the 3′-OH end where ‘A’ in Cq3 is replaced by ‘T’ in Cq4 and the rest of the sequences were unchanged and both could amplify a 380 bp corresponding band. Two PCR reactions were run in parallel; and in each of them, the template was 10 ng of single mosquito DNA. In one reaction, the primers Cq1, Cq2 and Cq3 were combined (10 pmol each) and in the other one Cq3 was replaced by Cq4. The reaction mixture contained 1 × PCR buffer, 200 µl dNTP mix and 2.5 U Taq Polymerase with a final reaction volume of 50 µl. The PCR conditions were 5 min at 94 °C for the first cycle followed by1 min at 94 °C, 2 min at 49 °C and 2 min for 72 °C for 29 cycles and 10 min at 72 °C for the final extension. All the chemicals used in this study were purchased from Sigma (Sigma-Aldrich Corporation, Bangalore, India), Roche (Roche Applied Science, Mannheim, Germany).

### Electrophoretic analysis of PCR products

DNA fragments contained in PCR products following PCR amplification were analysed by gel electrophoresis with 2% agarose gel, containing 0.5 mg/ml of Ethidium bromide (EtBr)^40^. The estimated band size of 274 bp unique for *Cx. quinquefasciatus* species was used for the authentication^41^. For genotypying of the VGSC gene, the characteristic 380 bp band corresponding to 1014F and 1014L specific primers of culex *quinquefasciatus* (Cq3 and Cq4 respectively) was used. Appearance of the 380 bp band in both the 1014L-specific primer and 1014F-specific primer in an individual mosquito indicated heterozygous condition (LF). The appearance of this band only in 1014L-specific primer (Cq4) indicated homozygous susceptible (LL) genotype and appearance in resistant specific primer (Cq3) indicated homozygous resistant (FF) genotype. The L1014Fkdr allelic frequency was calculated and tested for conformity to Hardy–Weinberg equilibrium within and between study sites.

### Statistical analyses

All statistical analysis were performed on SPSS version 22 software. Descriptive statistics were used to analyse bioassay results. CDC bioassay results were expressed as percentage mortality at recommended diagnostic time for the insecticides. Time response curves and knockdown times for 50% (KDT50) and 95% (KDT95) values of the test population were generated using Probit analysis. Analysis of variance was used to test for the significant difference amongst different insecticide exposures at study localities. 95% confidence interval (0.05) was used in testing of hypothesis. Hardy–Weinberg principle was used in the determination of allelic frequencies in mosquito populations, using the formula: f (F) = (2FF + LF)/2n to calculate the frequency of the L1014F kdr alleles in the population. Chi-square test (χ^2^) was employed for assessing the conformity or deviations from the Hardy–Weinberg equilibrium in the distribution of L1014F allele frequencies in different populations of the lymphatic filariasis vectors, using 95% (0.05) confidence level. Data analyzed were presented using tables and line graphs for ease of interpretation.

## Results

### Susceptibility studies

Female *Culex quinquefasciatus* mosquitoes from Emman Uruan resisted permethrin insecticide (total mortality of 18%) the most. They also showed resistance to alphacypermethrin (total mortality of 82%) but suspected resistance to deltamethrin insecticide (with total mortality of 90%) recorded for the same population of mosquitoes (Table 1). The mean mortality in the groups treated with deltamethrin and alphacypermethrin insecticides were significantly (*p* < 0.05) higher than those observed in the permethrin insecticide treated groups. The mean mortality in the groups treated with deltamethrin insecticides was significantly higher (*p* < 0.05) than what was observed in the groups treated with alphacypermethrin insecticide.

**Table 1:** Susceptibility bioassayon *Culex quinquefasciatus* population from Emman Uruan in Northern Uruan District, Uruan LGA, Akwa Ibom state, Nigeria to Pyrethroid Insecticides.

Insecticides	Number Exposed	Number of Replicates	Mean KD ± SD	Total KD at DT (KD in control at DT) (%)	Abbot’s corrected Mortality (%)	Status
EmmanUruan, Northern Uruan District						
Permethrin (21.5 µg/bottle)	100	4	4.50 ± 0.58	18 (4)	14.58	Resistant
Alphacypermethrin (12.5 µg/bottle)	100	4	20.50 ± 0.58^a^	82 (4)	81.25	Resistant
Deltamethrin (12.5 µg/bottle)	100	4	22.50 ± 0.58^a,b^	90 (4)	89.58	Suspected Resistance
IduUruan, Central Uruan District						
Permethrin (21.5 µg/bottle)	100	4	5.20 ± 0.50	21 (0)	NA	Resistant
Alphacypermethrin (12.5 µg/bottle)	100	4	21.00 ± 0.00^a^	84 (8)	81.25	Resistant
Deltamethrin (12.5 µg/bottle)	100	4	22.75 ± 0.50^a,b^	91 (0)	NA	Suspected Resistant
ItukMbang, Southern Uruan District						
Permethrin (21.5 µg/bottle)	100	4	5.75 ± 0.50	23 (0)	NA	Resistant
Alphacypermethrin (12.5 µg/bottle)	100	4	21.25 ± 0.50^a^	85 (4)	84	Resistant
Deltamethrin (12.5 µg/bottle)	100	4	23.25 ± 0.50^a,b^	93 (8)	92	Suspected Resistant

Number of mosquitoes per replicate = 25, a = *p* ≤ 0.05 (comparing other insecticide test groups with the permethrin), b = *p* ≤ 0.05 (comparing Alphacypermethrin with Deltamethrin).

Female *Culex quinquefasciatus* mosquitoes from IduUruan, showed high resistance (total mortality of 21%) to permethrin insecticide, they were also resistant to alphacypermethrin (total mortality of 84%). However, they showed only suspected resistance to deltamethrin, insecticide, (total mortalities of 91%) (Table 1). The mean mortality in the deltamethrin and alphacypermethrin insecticide treated groups were significantly (*p* < 0.05) higher than that observed in the permethrin treated group. The mean mortality in the group treated with deltamethrin insecticide was significantly (*p* > 0.05) higher, compared to what was observed in the mean mortality of the *Culex quinquefasciatus* mosquito population treated alphacypermethrin insecticide.

Female *Culex quinquefasciatus* mosquitoes from Ituk Mbang showed high resistance (total mortality of 23%) to permethrin. They equally showed resistance to alphacypermethrin insecticide (total mortality of 85%), but only suspected resistance to deltamethrin insecticide (total mortality of 93%) (Table 1). The mean mortality in the deltamethrin and alphacypermethrin insecticide treated groups were significantly (*p* < 0.05) higher than that observed in the permethrin insecticide treated group. The mean mortality in the *Culex quinquefasciatus* group treated with deltamethrin insecticide, was significantly (*p* < 0.05) higher compared to what was observed in the alphacypermethrin treated group.

Summary of results of mortality and susceptibility status of *Culex quinquefasciatus* vector population in the three different Study sites exposed to three different pyrethroid insecticides are presented in Table 2.

**Table 2:** Summary of results of mortality and susceptibility status of *Culex quinquefasciatus* vector population in the three different Study sites exposed to three different pyrethroidinsecticides.

Study sites	Permethrin			Alphacypermethrin			Deltamethrin		
	N	%M	Status	N	%M	Status	N	%M	Status
Emman Uruan	100	18	R	100	82	R	100	90	RS
Idu Uruan	100	21	R	100	84	R	100	91	RS
Ituk Mbak	100	23	R	100	85	R	100	93	RS

N, Number of mosquitoes exposed; %M, Percentage mortality; R, Resistant; RS, Resistance Suspected.

### Knock down effect

**Figure 2:**
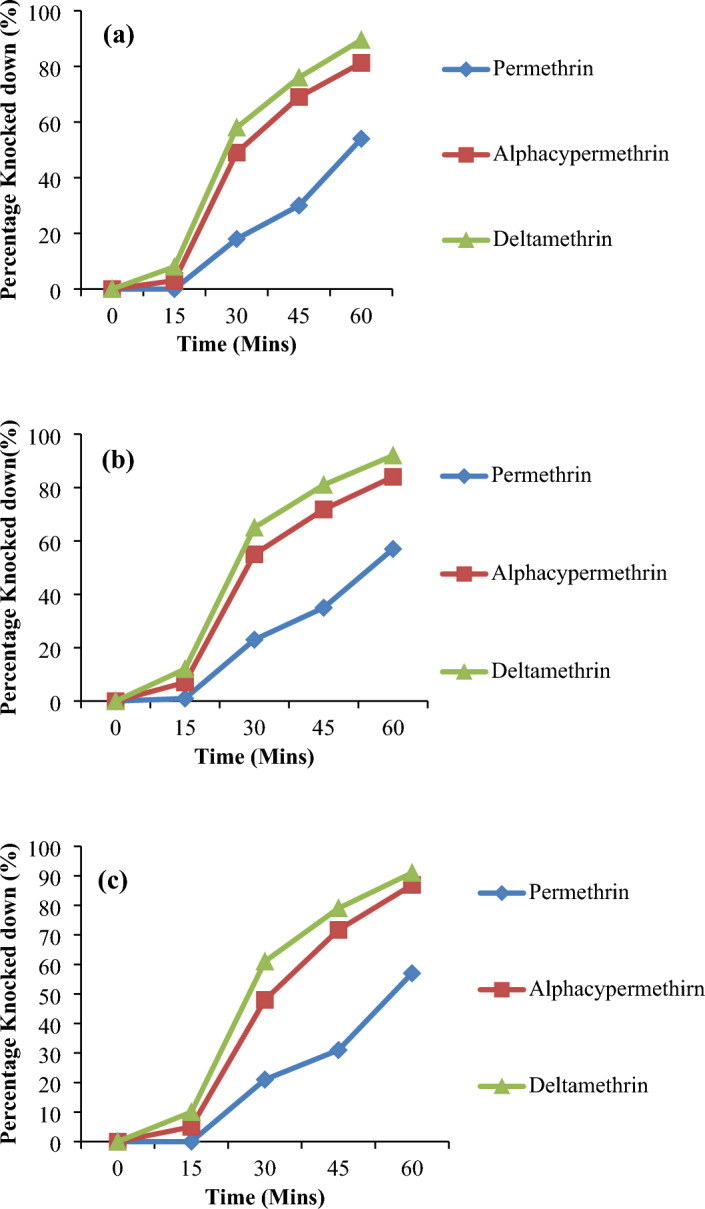
Knockdown rate of vector populations from (**a**) Emman Uruan (**b**) Ituk Mbang and (**c**) Idu Uruan exposed to three different pyrethroid insecticides.

The results of knockdown assessment determined over a one-hour exposure period of female *Culex quinquefasciatus* mosquitoes from the different study sites to three different pyrethroid insecticide are presented in Fig. 2a–c. Results indicate that in all study sites, knockdown was more rapid for deltamethrin and alphacypermethrin than for permethrin. Noteworthy is the observation that all mosquito populations from the different study sites showed over 90% knock-down within the one-hour exposure to deltamethrin and alphacypermethrin. Exposure times which resulted in 50% and 95% knockdown (KDT50 and KDT95) estimated for each insecticide using a log-time probit model are presented in Table 3. The median knockdown time (KDT50) ranged from 50.50 to 55.02 min while the KDT95 ranged from 144.62 to 154.52 min for permethrin; for deltamethrin, KDT50 ranged from 26.72 to 36.43 min and KDT95 from 65.44 to 68.08 min; and for alphacypermethrin, KDT50 ranged from 31.46 to 32.73 min and KDT95 from 76.67 to 81.9 min across the study sites. Among these pyrethroids, deltamethrin, and alphacypermethrin were more effective in their toxicity on the lymphatic filariasis vectors than permethrin considering their KDT50 and KDT95 value.

**Table 3:** Knock down times (KDTs) for the vector populations in the three different study sites after exposure to different insecticides.

Study sites	Number exposed	Knock down time (Minutes)					
		Permethrin		Alphacypermethrin		Deltamethrin	
		KDT50 (95% CL)	KDT95 (95% CL)	KDT50 (95% CL)	KDT95 (95% CL)	KDT50 (95% CL)	KDT95 (95% CL)
Emman Uruan	100	55.02 (38.89–627.78)	144.62 (77.67–7489647.58)	32.73 (30.21–35.28)	76.67 (67.16–91.74)	28.08 (25.70–30.41)	68.08 (59.84–80.86)
Ituk Mbang	100	0.50 (53.89–48.76)	154.52 (117.96–238.70)	31.46 (21.09–42.18)	81.95 (55.64–301.83)	26.72 (24.38–28.99)	65.44 (57.55–77.64)
Idu Uruan	100	55.02 (39.89–627.78)	144.62 (77.67–7489647.58)	32.734 (30.21–35.28)	76.67 (67.16–91.74)	28.08 (25.70–30.41)	68.08 (59.84–80.86)

*KDT50* knockdown time for 50% mosquitoes, *KDT95* knockdown time for 95% mosquitoes, *CL* Confidence limit.

### Molecular identification of the vectors

**Figure 3:**
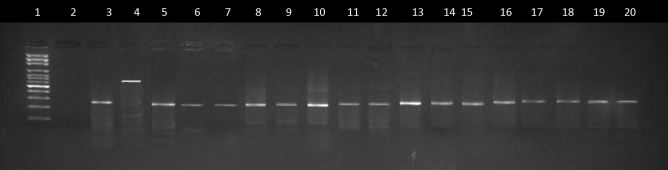
UV illuminated Agarose gel showing DNA fragments bands of PCR amplification for the authentication ofthe *Culex quinquefasciatus* species from study sites used for this study. Lane 1: 100-basepair DNA size marker ladder. Lane 2: Negative control. Lanes 3: *Culex quinquefasciatus* positive control (274 bp). Lane 4: *Culex pipens* positive control (610 bp). Lanes 5–20: *Culex quinquefasciatus* (wild samples).

Figure 3 shows the ultraviolet light illuminated agarose gel showing PCR amplified DNA fragments bands for the establishment of the molecular identity of the vector populations from study sites. 100% of the mosquitoes randomly sampled were identified as *Culex quinquefasciatus* as revealed by the size of their ITS 2 DNA fragment electrophoretic band being 274 base pair.

### Genotypes and the kdrallelic frequencies of LF vectors, Culex quinquefasciatus

**Figure 4:**
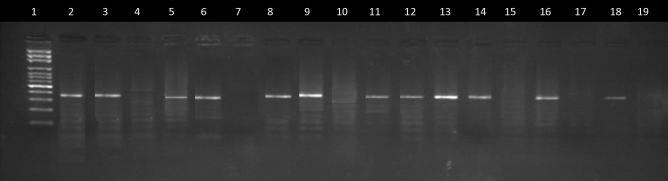
UV illuminated Agarose gel showing DNA fragments bands of PCR amplification for AS-PCR identification of *kdr* alleles in *Culex quinquefasciatus* from study sites. Two PCR reactions were run in parallel for each specimen. Lane 1: 100-basepair DNA size marker ladder; Lanes 2, 3 and 4 are for Cq3 positive control, Cq4 Positive control and negative control respectively. Lanes 5 and 6 for specimen 1; lanes 7 and 8 for specimen 2. Genotype results: Lanes 7 and 8, 15 and 16, 17 and 18: Susceptible (LL) specimens lacking the L1014F mutation; Lanes 9 and 10: Homozygous resistant (FF) specimens with L1014F mutation; Lanes 5 and 6, 11 and 12, 13 and 14: Heterozygous resistant (LF) specimens with L1014F mutation.

The detection of the L1014F *kdr* allele (*kdr*-w type) in a subsample of *Culex quinquefasciatus* populations which comprised of both susceptible population (those that didn’t survive the test) and resistant population (those that survived the test) revealed that the mutation was present in the population of mosquitoes studied. Figure 4 shows the gel electrophoresis bands of AS-PCR products used for the genotyping. The results revealed that only 18.6% of the mosquitoes possessed susceptible (LL) genotype, 36.3% and 45.1% had heterozygous (LF) and homozygous (FF) genotypes respectively. The frequency of the resistant phenotypes ranged from 35.14 to 55.3% across the study sites with a net of 45.1% resistant phenotype recorded in this study (Table 4). The 1014F allelic frequency calculated from Hardy–Weinberg principle for vector populations across the study sites ranged from 0.500 (50.00%) to 0.7763 (77.63%) (Table 5). Chi-square analysis of the allelic frequencies distribution to ascertain deviations from the Hardy–Weinberg equilibrium by vector populations at different sites are presented. All the Vector populations witnessed significant (*p* < 0.05) deviation from the Hardy–Weinberg equilibrium with their calculated chi square value being higher than the tabulated chi square value (probability of 0.05 and df of 1).

**Table 4:** Genotypic and Phenotypic frequencies of the mutations in the voltage gated sodium channel gene of *C. quinquefasciatus* from three different study sites in Uruan LGA of Akwa Ibom State.

Study sites	Number sampled	Genotype frequency (%)			Phenotype frequency (%)	
		FF	LF	LL	Resistant	Susceptible
EmmanUruan	38	17 (44.7)	13 (34.2)	8 (21.1)	17 (44.7)	21 (55.3)
ItukMbang	37	13 (35.14)	11 (29.72)	13 (35.14)	13 (35.14)	24 (64.86)
IduUruan	38	21 (55.3)	17 (44.7)	0 (0.0)	21 (55.3)	17 (44.7)
Total	113	51 (45.1)	41 (36.3)	21 (18.6)	51 (45.1)	62 (54.9)

FF denotes the homozygous resistant L1014F genotype, LF denotes the heterozygous L1014F genotype and LL denotes the homozygous susceptible wild genotype.

**Table 5:** Chi-square analysis for the conformity of the L1014F mutant alleles of the voltage gated sodium channel in *C. quinquefasciatus* from three different study sites in Uruan LGA of Akwa Ibom State to Hardy–Weinberg equilibrium.

Study sites	N	Allelic frequencies in the parent’s population		Observed genotypic frequencies for the next generation			Expected genotypic frequencies for the next generation			χ^2^_cal_	df	χ^2^_tab_
		f(F)	f(L)	f(FF)	f(LF)	f(LL)	f(FF)	f(LF)	f(LL)			
Eman Uruan	38	0.6184	0.3816	0.3824	0.4719	0.1456	14.5329	17.9342	5.5329	36.0263	1	3.841
Ituk Mbang	37	0.5000	0.5000	0.2500	0.5000	0.2500	9.2500	18.500	9.25	35.0270	1	3.841
Idu Uruan	38	0.7763	0.2237	0.6027	0.4648	0.1349	22.9013	13.1974	1.9013	36.0263	1	3.841

FF denotes the homozygous resistant L1014F genotype, LF denotes the heterozygous L1014F genotype and LL denotes the homozygous susceptible wild genotype.

## Discussion

Although *Culex quinquefasciatus* mosquitoes are predominant in most cities across Sub-Saharan Africa and they are of major epidemiological significance as vectors of important diseases like West Nile Virus and filariasis, susceptibility of different populations of these vectors to pyrethriod insecticides has not been widely investigated. Furthermore, the presence and frequency of resistance alleles in the local populations has been rarely investigated in mosquito populations from AkwaIbom State^30^. Monitoring is an integral part of any resistance management strategy, which allows informed decisions about choice of insecticides^30^.

The tested populations from the three study sites of the study area exhibited very high resistance against permethrin with knocked down rates being as low as 18%. Although the mortality/knocked down rates in the mosquito populations were significantly higher with exposure to deltamethrin and alphacypermethrin insecticides, no susceptibility status was recorded in this present study as none of the populations exposed to these insecticides had up to 98% mortality. The observations in this present study is contrary to the susceptibility status recorded in *Anopheles gambiae* populations by Opara et al.^43^.The mosquitoes exposed to pyrethroid insecticides were highly susceptible in this study. Nevertheless, the results obtained in the current work are in line with previous bioassay records in Culex populations from the city of Yaoundé including *Culex quinquefasciatus*, *Cx. duttoni*, *Cx. antennatus*, *Cx. perfuscus* and *Cx. tigripes* specimens^44^. In addition, the results obtained in this current work is in tandem with the bioassay records on *Anopheles gambiae* populations from crude oil impacted coastal areas in Akwa Ibom State exposed to the same set of pyrethriod insecticides^28^. The low level of susceptibility to pyrethroids is of major concern with potential operational relevant implications in the control and elimination of diseases transmitted by these vectors.

Presence and frequency analyses of target-site resistance mutations recorded the kdr allele (L1014F) at high frequencies in all populations. This was contrary to the low kdr allelic (L1014F) frequency recorded in the work of^30^. The observation that these populations of the vector witnessed deviation from the Hardy–Weinberg equilibrium in the distribution of their resistant allelic frequencies calls for serious concern. According to Andrews^45^ on explanation in Hardy–Weinberg principle, such observation implies that these populations will constantly experience evolution of these resistant mosquitoes as a result of the mutation that is ongoing. Hence, the tendency of natural selection taking out the susceptible populations in the same study sites over time cannot be ruled out. The high level of resistance recorded in this study was strongly corroborated by the trend in the median knockdown time (KDT_50_) observed. In this study, a significance difference in the insecticide resistance capacity of the vectors was observed between permethrin and the other two pyrethroids. Knock down was more rapid for the type II pyrethroids than for the type I pyrethroid as reflected by the trend in KDT_50_. The type II pyrethroids including deltamethrin and alphacypermethrin insecticides were more effective on the vectors across the study sites than the type I pyrethriod, permethrin as reflected by the fact that with permethrin, time taken taken for 50% of the population to be knock down was very high. Similar observations have been reported^46,47^ on the toxicity potentials of type 1 and type II pyrethroids. It has been explained^46^ that the type II pyrethroids have a cyano group at the α-benzylic position (the α-carbon of the 3-phenoxybenzyl alcohol) which cause a pronounced convulsive Phase that results in ‘better kill’ due to depolarization of the nerve axons and terminals that are irreversible. In addition, the differing toxicity effects have been explained by the fact that the duration of modified sodium currents by type I compounds lasts only tens or hundreds of milliseconds, while those of type II compounds last for several seconds or longer^46^.

The current pyrethroid resistance recorded in this work and previous works may have derived from the increased selection pressure induced by the massive deployment of pyrethroid treated nets across the country in conjunction with the use of pyrethroid-based insecticides in agricultural pest control^30^. In addition to insecticides, xenobiotics could also induce high resistance in this mosquito species due to its preference for organically polluted habitats at the larval stage^48^. The influence of organic pollution on *Cx. quinquefasciatus* susceptibility to insecticide has so far not been adequately explored.

## Conclusion

This work has successfully provided information on susceptibility status of *Cx. quinquefasciatus* to commonly used pyrethroid insecticides as well as detected L1014F kdr alleles in the vector populations. The CDC bioassay results showed that vector populations from Uruan LGA were resistantor had potentials of becoming resistant to pyrethriod insecticides used. High frequency of L1014F kdr alleles have been recorded across vector populations in all the study sites. It can then be concluded that point mutation (TTA–TTT) in the vgsc genes (L1014F alleles) resulting in leucine amino acid being substituted with phenylalanine amino acid at residue 1014 of the voltage gated sodium channel may have contributed immensely to the insecticide resistance recorded in the vector populations from these areas. Findings from Hardy–Weinberg analysis revealed the tendency of recording an entire population of resistant vectors in these study areas over time due to natural selection. It is recommended that further investigation should be carried out in order Lymphatic filariasis endemic communities of the state, for informed decisions on the choice of insecticides.

## Data Availability

The data sets in this study are available from the corresponding author on reasonable request.

## Abbreviations

AS-PCR: Allele-specific polymerase chain reaction
CDC: Centre for disease control
DD: Discriminating doses
DT: Diagnostic time
EtBr: Ethidium bromide
IVM: Integrated vector management
Kdr: Knockdown resistance
KDT: Knock down time
LF: Lymphatic filariasis
MBDs: Mosquito-borne diseases
MDA: Mass drug administration
NMEP: National Malaria Elimination Programme
PCR: Polymerase chain reaction
TAE: Tris–Acetate ethylene diamine tetra acetic acid
VGSC: Voltage-gated sodium channel
WHO: World Health Organization
